# Outcomes of Trabeculo-Descemet's Membrane Microperforation During Combined Phacoemulsification and Deep Sclerectomy

**DOI:** 10.18502/jovr.v20.15249

**Published:** 2025-07-30

**Authors:** Mahdi Sharifzadeh Kermani, Sana Jalalmanesh, Hesam Fat'hi-zadeh, Ali Sharifi, Amin Zand

**Affiliations:** Clinical Research Development Unit, Shafa Hospital, Kerman University of Medical Sciences, Kerman, Iran

**Keywords:** Deep Sclerectomy, Intraocular Pressure, Microperforation, Open-angle Glaucoma, Pseudoexfoliation, Trabeculo-Descemet's Membrane

## Abstract

**Purpose:**

To evaluate the impact of inadvertent intraoperative microperforation of the trabeculo-Descemet's membrane (TDM) on postoperative outcomes in cases with cataract and either primary open-angle glaucoma (POAG) or pseudoexfoliative glaucoma (PEXG) during combined phacoemulsification and deep sclerectomy (PDS).

**Methods:**

In this prospective case–control study, 73 eyes of 73 patients with cataract and POAG/PEXG who underwent PDS were enrolled. Patients were divided into two groups based on the presence or absence of inadvertent intraoperative TDM microperforation. The primary outcome measures included intraocular pressure (IOP), number of antiglaucoma drugs, and assessment of adverse effects during a six-month follow-up period.

**Results:**

Significant decreases in IOP were observed at all follow-up time points (day 7, month 1, and month 6) in both groups compared to baseline (P < 0.001). No significant differences in IOP were found between the groups at any time point, except on day 7, while the perforation group exhibited a significantly lower IOP compared to the non-perforation group (11.84 ± 4.78 vs 14.03 ± 2.50 mmHg, P = 0.017). At month 6, the mean number of antiglaucoma drugs was 0.19 ± 0.46 in the perforation group and 0.06 ± 0.23 in the non-perforation group, suggesting a significant reduction in both groups compared to baseline (P < 0.001) with no statistically significant difference between the two groups (P = 0.124). Hypotony was observed in 3 (8.1%) eyes with perforation, but it resolved without persistent complications in subsequent follow-ups.

**Conclusion:**

Inadvertent TDM microperforation during PDS for POAG or PEXG did not impact IOP outcomes compared to non-perforated cases over a six-month period, nor did it increase the incidence of complications.

##  INTRODUCTION

Deep sclerectomy (DS) stands as a well-established and safe non-penetrating surgical intervention for glaucoma. It effectively reduces intraocular pressure (IOP) with fewer complications compared to trabeculectomy.^[1]^ The primary resistance to aqueous humor outflow resides within the inner wall of Schlemm's canal, particularly in the juxtacanalicular network. During DS, these structures are meticulously dissected with a deep flap created beneath a surface flap, thereby preserving the integrity of Descemet's window, also known as the trabeculo-Descemet's membrane (TDM). This membrane facilitates aqueous drainage while preventing excessive filtration.^[2]^ Additionally, it serves to regulate IOP and safeguard against a sudden decline in postoperative IOP.^[3,4,5,6]^

The success of DS depends on the functionality of the TDM, which ensures adequate aqueous flow. However, inadvertent TDM perforations may occur, particularly during the learning curve of the procedure.^[7,8]^ These perforations, depending on their size, are generally categorized into microperforations and macroperforations. Large-scale perforations, known as macroperforations, can lead to uncontrolled aqueous humor outflow, potentially resulting in ocular hypotony and other related complications, such as choroidal effusion or iris incarceration.^[9]^ Some previous studies have revealed a 30% conversion rate to trabeculectomy following the occurrence of these types of TDM perforations during DS.^[10]^ In DS, microperforation refers to a controlled, small-scale breach of the TDM during surgery, allowing gradual aqueous drainage through the trabeculo-Descemet's window and aiding in IOP reduction.^[7,9,11,12]^ Some previous studies have shown that perforation of the TDM during the DS procedure may be associated with a lower incidence of postoperative complications, without significant differences in postoperative IOP outcomes in eyes with primary congenital glaucoma.^[9]^ Conversely, other studies have indicated that in eyes with POAG, combined phacoemulsification and deep sclerectomy (PDS), when modified by TDM microperforations, may significantly decrease IOP at medium-term follow-up.^[11]^ In short, there are discrepancies in the observed outcomes of TDM microperforation, and no comparative case–control study has evaluated the precise outcomes of these microperforations during the PDS procedure. Therefore, this case–control study aims to investigate the impact of inadvertent TDM microperforations during PDS on postoperative outcomes in eyes with cataract and either primary open-angle glaucoma (POAG) or pseudoexfoliative glaucoma (PEXG).

##  METHODS

### Study Design

This prospective case–control study was carried out at the Ophthalmology Department of Shafa Hospital in Kerman, Iran, from June 2022 to October 2023. The study adhered to the ethical guidelines outlined in the Declaration of Helsinki, and all participants provided written informed consent. The study protocol received approval from the Ethics Committee of Kerman University of Medical Sciences (Ethics Code: IR.KMU.AH.REC.1401.169).

### Subjects

Seventy-three eyes of 73 cases diagnosed with clinically notable cataracts accompanied by either POAG or PEXG were enrolled in this prospective comparative study. The inclusion criteria comprised an IOP of 22 mmHg or higher, evidence of glaucoma progression despite the use of antiglaucoma medications, or nonadherence to these medications. In cases of bilateral disease, the eye with more severe glaucoma was selected for enrollment. These patients underwent PDS surgery, and eyes with inadvertent microperforation of the TDM during the surgery were compared to eyes without this complication. Microperforation was defined as a controlled, small-scale perforation, approximately the size of a 30-gauge needle, that occurred in the TDM during the surgical procedure and did not require further intervention.^[9,11,13]^

The exclusion criteria included angle-closure glaucoma, secondary open-angle glaucoma caused by factors other than PEXG, previous intraocular surgery or trauma, corneal edema or opacity, other intraoperative complications such as complicated cataract surgery, and inadequate compliance with the instillation of antiglaucoma medication or macroperforation of the TDM. Individuals with a follow-up period of less than six months or under the age of 18 were also excluded.

### Surgical Procedure

A single surgeon (MSK) performed all procedures using a uniform phacoemulsification technique, which involved implanting a one-piece hydrophobic acrylic intraocular lens (enVista, Bausch & Lomb, Bridgewater, NJ, USA). A temporal corneal incision was made for lens implantation.

The DS procedure commenced with corneal traction sutures to expose the superior quadrant, followed by a limbal conjunctival incision. Subconjunctival mitomycin C (0.25 mg/ml) was applied for 2 minutes using sponges. A 5 × 5 mm superficial scleral flap was fashioned, beneath which a 4 × 4 mm deeper flap was created. Dissection of the deep flap continued to the Schlemm's canal, where spontaneous aqueous percolation was observed. The dissection was extended up to 1.5 mm into the clear cornea. The deep flap was excised, and the superficial flap was loosely secured at the corners with two 10-0 nylon sutures. The conjunctiva was then closed using 10-0 nylon stitches.^[3,7]^

### Evaluation of Outcomes

All glaucoma medications were halted on the first day following surgery. Patients were treated with topical ciprofloxacin four times daily for one week and betamethasone four times daily for two weeks, with a gradual tapering of the topical steroid over a month. Postoperative evaluations were conducted on days 1 and 7, as well as at months 1 and 6. The day 1 assessment focused on identifying early postoperative complications, including hypotony, macroscopic hyphema, a shallow or flat anterior chamber, and choroidal effusion or hemorrhage. Outcome analysis included detailed ophthalmic assessments on day 7 and at the one- and six-month follow-up visits.

IOP measurements were obtained by a single observer (SJ) using a Goldmann applanation tonometer. Best-corrected visual acuity (BCVA) was measured at all follow-up visits, and logMAR values were used for statistical analysis of visual acuity. Complications were also monitored at each follow-up appointment. Ocular hypotony was defined as IOP ≤ 5 mmHg, based on postoperative criteria,^[14]^ while a shallow anterior chamber was identified according to the criteria established by Teehasaenee and Ritch.^[15]^

The surgery was considered completely successful if the IOP ranged from 6 to 20 mmHg with at least a 30% reduction in IOP without the use of glaucoma medications or further surgery, compared to preoperative values.^[16,17]^ Partial (qualified) success was defined as an IOP of 6–20 mmHg and a 20–30% reduction in IOP achieved with antiglaucoma medications. When necessary, β -blockers (such as Timolol) and carbonic anhydrase inhibitors (acetazolamide or dorzolamide) were the first-line medications for postsurgical management. Failure was identified when neither criterion for success was met, further glaucoma surgery was required, or there was a loss of light perception.17

### Statistical Analysis 

Data analysis was performed using SPSS version 29 (IBM Corp., Armonk, NY, USA). Continuous variables were expressed as mean ± standard deviation (SD), and categorical variables were reported as percentages. Intergroup comparisons were made using the independent samples t-test and chi-square test, while within-group comparisons of baseline and follow-up data were assessed using the paired samples t-test. A P-value of < 0.05 was considered statistically significant.

##  RESULTS

### Subject Dispositions 

A total of 73 eyes from 73 individuals were divided into two groups based on the presence or absence of TDM microperforation. Among them, 37 eyes experienced inadvertent intraoperative TDM microperforation, while 36 eyes did not encounter this complication. At baseline, no significant differences were found between the perforation and non-perforation groups regarding age (69.24 ± 8.48 vs. 69.81 ± 6.69 years, respectively, P = 0.755), BCVA (1.06 ± 0.86 vs. 1.12 ± 0.81 logMAR, respectively, P = 0.774), IOP (20.62 ± 4.81 vs. 19.17 ± 5.53 mmHg, respectively, P = 0.234), number of antiglaucoma medications (1.38 ± 1.04 vs. 1.00 ± 0.93, respectively, P = 0.105), or distribution of POAG (70.3% vs. 58.3%, respectively, P = 0.334) [Table 1].

### Intraocular Pressure and Antiglaucoma Medications 

Both groups showed a significant decrease in IOP at all follow-up time points when compared to baseline (P < 0.001). The average IOP was significantly lower in the perforation group than in the non-perforation group on day 7 (11.84 ± 4.78 vs. 14.03 ± 2.50 mmHg, respectively, P = 0.017), with no significant differences observed at the other time points. The mean IOP reduction at six months compared to baseline was similar between the two groups and was not statistically significant (P = 0.656). Similarly, the mean number of antiglaucoma drugs decreased significantly in both groups at six months compared to baseline (P < 0.001), with no significant difference between the perforation and non-perforation groups (P = 0.124) [Tables 2 & 3].

We found that overall success was attained in 27 eyes (73.0%) in the perforation group and 22 eyes (61.1%) in the non-perforation group (P = 0.326). Complete success was observed in 17 eyes (45.0%) from the perforation group and 14 eyes (38.9%) from the non-perforation group. Additionally, 10 eyes (27.0%) in the perforation group and 8 eyes (22.2%) in the non-perforation group achieved qualified success. Failures, defined as not meeting the criteria for at least qualified success, occurred in 10 eyes (27.0%) in the perforation group and 14 eyes (38.9%) in the non-perforation group during follow-up (P = 0.558) [Table 3]. Importantly, neither group required further glaucoma surgery or experienced loss of light perception during the follow-up period.

### Visual Acuity

Both groups exhibited a significant increase in BCVA at six months relative to their preoperative levels (P < 0.001), with no significant difference in mean BCVA between the two groups at this follow-up (P = 0.517) [Table 3].

**Table 1:** Baseline patients and ocular characteristics

**Variable**	**Perforation (** ***n*** ** = 37)**	**Non-perforation (** ***n*** ** = 36)**	***P*** **-value** * *
Age (yrs), mean ± SD	69.24 ± 8.48	69.81 ± 6.69	0.755
Gender, *n* (%)	Female: 20 (54.1)	Female: 19 (47.2)	0.642
	Male: 17 (45.9)	Male: 17 (52.8)	
BCVA (logMAR), mean ± SD	1.06 ± 0.86	1.12 ± 0.81	0.774
IOP (mmHg), mean ± SD	20.62 ± 4.81	19.17 ± 5.53	0.234
Anti-glaucoma medications (*n*), mean ± SD	1.38 ± 1.04	1.00 ± 0.93	0.105
Glaucoma type, *n* (%)	POAG: 26 (70.3)	POAG: 21 (58.3)	0.334
	PEXG: 11 (29.7)	PEXG: 15 (41.7)	
BCVA, best-corrected visual acuity; IOP, intraocular pressure; logMAR, logarithm of the minimum angle of resolution; *n*, number; PEXG, pseudoexfoliative glaucoma; POAG, primary open-angle glaucoma; SD, standard deviation			

**Table 2:** IOP at baseline and follow-up visits

**IOP (mmHg), mean ± SD (range)**	**Perforation (** ***n*** ** = 37)**	**Non-perforation (** ***n*** ** = 36)**	***P*** **-value** * *
Baseline	20.62 ± 4.81	19.17 ± 5.53	0.234
Day 7	11.84 ± 4.78	14.03 ± 2.50	0.017
Month 1	13.27 ± 3.29	13.47 ± 2.32	0.764
Month 6	13.53 ± 2.11	12.91 ± 1.90	0.204
*n*, number; SD, standard deviation; IOP, intraocular pressure			

**Table 3:** Surgical outcomes at month 6

**Outcome**	**Perforation (** ***n*** ** = 37)**	**Non-perforation (** ***n*** ** = 36)**	***P*** **-value**
Overall Success, *n* (%)	27 (73.0)	22 (61.1)	0.326
Complete Success, *n* (%)	17 (45.9)	14 (38.9)	0.558
Qualified Success, *n* (%)	10 (27.0)	8 (22.2)	
Failure, *n* (%)	10 (27.0)	14 (38.9)	
IOP reduction (%), mean ± SD	29.38 ± 23.74	26.93 ± 22.36	0.656
Anti-glaucoma medications (*n*), mean ± SD	0.19 ± 0.46	0.06 ± 0.23	0.124
BCVA (logMAR), mean ± SD	0.31 ± 0.45	0.37 ± 0.30	0.517
BCVA, best-corrected visual acuity; IOP, intraocular pressure; logMAR, logarithm of the minimum angle of resolution; *n*, number; SD, standard deviation			

**Table 4:** Postoperative complications

**Complication**	**Perforation (** ***n*** ** = 37)**	**Non-perforation (** ***n*** ** = 36)**	***P*** **-value**
Hypotony (IOP ≤ 5 mmHg)	3 (8.1)	0	0.240
Macroscopic hyphema	1 (2.7)	1 (2.8)	1.000
Peripheral anterior synechiae	0	0	1.000
Shallow/flat AC	0	0	1.000
Choroidal effusion/hemorrhage	0	0	1.000
Infectious endophthalmitis	0	0	1.000
AC, anterior chamber; IOP, intraocular pressure; n, number			

### Complications and Adverse Events 

On day 1, hypotony was observed in three eyes (8.1%) in the perforation group, while no cases were reported in the non-perforation group (P = 0.240). However, all eyes had IOP > 5 mmHg in subsequent follow-ups. Macroscopic hyphema was observed in one eye in each group on day 1, resolving by day 7. No other major complications—including peripheral anterior synechiae, shallow/flat anterior chamber, or choroidal effusion/hemorrhage—were reported in either group during follow-ups [Table 4].

##  DISCUSSION

Non-penetrating DS surgery stands as a preferred option for managing glaucoma. This is due to its ability to regulate aqueous humor filtration without inducing early postoperative hypotony, which is commonly observed following trabeculectomy.^[18]^ Furthermore, the long-term safety and efficacy of performing phacoemulsification cataract surgery in combination with non-penetrating DS as a primary glaucoma procedure are well-established.^[19,20]^ However, inadvertent microperforation of the TDM remains a common complication during non-penetrating glaucoma surgeries, including traditional DS, and it could potentially necessitate conversion to trabeculectomy. Notably, microperforations do not impede the completion of the operation.^[21]^ Regardless of the occurrence of perforation, the overall success rates in our study were lower than those reported in some previous studies.^[19,20]^ These differences may be due to various factors such as a narrower definition of qualified success, a broader definition of failure, and higher baseline IOP levels in our study compared to similar studies.

Previous studies have demonstrated the consequences of microperforations in significantly reducing IOP.^[22]^ TDM microperforations may also occur during other ocular surgeries, such as phacoemulsification, viscocanalostomy, and cyclophotocoagulation, resulting in a reduction in IOP.^[23,24,25,26]^ The mechanisms underlying IOP reduction following TDM perforations include trans-scleral flow, activation of nonfunctional areas of Schlemm's canal, and uveoscleral outflow.^[23]^ Consequently, microperforations present a potential long-term solution for glaucoma management.^[11]^ Dagres et al^[26]^ evaluated the long-term outcome of phacoemulsification combined with viscocanalostomy that was complicated by the perforation of TDM during surgery. They found no significant difference in postoperative IOP and the number of antiglaucoma medications between the perforated and non-perforated groups at one week and later follow-ups. They detected that hypotony (IOP < 5 mmHg) was much more frequent in the perforated group compared to the non-perforated group (40% vs. 8.7%) at one day postoperatively. Still, it did not persist at subsequent follow-up visits. In another study, Elbably et al^[18]^ assessed the role of a porous collagen implant (Ologen) in DS, with and without TDM rupture, in eyes with open-angle glaucoma. They detected no discernible difference in the IOP reduction between the two groups at the last follow-up, one year postoperatively. Pullig et al^[27]^ assessed the potential benefits of the Descemet's window opening in canaloplasty. They demonstrated that intraoperative planned puncture of the Descemet's window tended to yield better results, although not statistically significant, in terms of IOP reduction, BCVA improvement, and postoperative medication use at one year. Furthermore, they reported minimal intra- and postoperative complications associated with this technique. Other studies reported that microperforations during traditional DS surgery were not associated with serious postoperative complications, including severe hypotony or hypotony-related complications.^[7,11,12,21]^ In this study, we found IOP reduction was significantly lower in eyes with TDM microperforation on day 7 after surgery in comparison to eyes without TDM microperforation. In our research, similar to the aforementioned studies, we found that IOP reduction during longer follow-ups (at one and six months), the need for postoperative antiglaucoma medications, and severe postoperative complications did not differ between eyes with and without TDM microperforation during the DS surgery.

Some authors have modified the DS surgical method by introducing various techniques, such as removing a portion of the scleral flap, in an effort to extend filtration in DS.^[5,28]^ Furthermore, some investigators have shown that penetrating DS—following standard dissection of the scleral flaps, anterior chamber penetration through the TDM, and iridectomy—may control intraoperative perforation of the TDM and offer long-term satisfactory outcomes, including sustained IOP reduction.^[24]^ Some studies have shown comparable long-term efficacy and safety between penetrating DS and the non-penetrating alternative.^[29,30,31]^ Kalala et al^[29]^ showed that the occurrence of serious postoperative complications, such as iris incarceration, choroidal effusion, and hypotony, decreased significantly in penetrating DS compared to traditional DS and trabeculectomy.

Our study had a few limitations, the most significant being the relatively short six-month follow-up period, accompanied by limited patient visits during that time. As a result, some complications may not have been fully captured, potentially impacting the detection of adverse effects related to TDM microperforations. This limitation was primarily due to the characteristics of the patient population, many of whom were referred from distant areas, making it difficult to arrange extended and more frequent follow-up visits. Additionally, we did not document certain baseline ocular characteristics, such as cataract type and severity, glaucoma stage, and visual field data, nor did we perform anterior segment OCT imaging for a detailed assessment of the angle and Descemet's membrane.

In summary, our findings suggest that inadvertent TDM microperforation during PDS surgery for POAG or PEXG does not significantly impact IOP outcomes or the need for antiglaucoma medications over six months. Moreover, intraoperative microperforation of the TDM during PDS was not associated with a higher incidence of postoperative complications. Further studies with more extended follow-up periods and more frequent visits are warranted to better understand the long-term implications of TDM microperforations in PDS surgery.

##  Financial Support and Sponsorship

None.

##  Conflicts of Interest

None.
